# HSPMdb: a computational repository of heat shock protein modulators

**DOI:** 10.1093/database/baaa003

**Published:** 2020-02-21

**Authors:** Prashant Singh, Breezy Unik, Anuradhika Puri, Gandharva Nagpal, Balvinder Singh, Ankur Gautam, Deepak Sharma

**Affiliations:** Council of Scientific and Industrial Research-Institute of Microbial Technology, Sector 39A, Chandigarh-160036, India

## Abstract

Heat shock proteins (Hsp) are among highly conserved proteins across all domains of life. Though originally discovered as a cellular response to stress, these proteins are also involved in a wide range of cellular functions such as protein refolding, protein trafficking and cellular signalling. A large number of potential Hsp modulators are under clinical trials against various human diseases. As the number of modulators targeting Hsps is growing, there is a need to develop a comprehensive knowledge repository of these findings which is largely scattered. We have thus developed a web-accessible database, HSPMdb, which is a first of its kind manually curated repository of experimentally validated Hsp modulators (activators and inhibitors). The data was collected from 176 research articles and current version of HSPMdb holds 10 223 entries of compounds that are known to modulate activities of five major Hsps (Hsp100, Hsp90, Hsp70, Hsp60 and Hsp40) originated from 15 different organisms (i.e. human, yeast, bacteria, virus, mouse, rat, bovine, porcine, canine, chicken, *Trypanosoma brucei* and *Plasmodium falciparum*). HSPMdb provides comprehensive information on biological activities as well as the chemical properties of Hsp modulators. The biological activities of modulators are presented as enzymatic activity and cellular activity. Under the enzymatic activity field, parameters such as IC_50_, EC_50_, DC_50_, *K_i_* and *K*_D_ have been provided. In the cellular activity field, complete information on cellular activities (percentage cell growth inhibition, EC_50_ and GI_50_), type of cell viability assays and cell line used has been provided. One of the important features of HSPMdb is that it allows users to screen whether or not their compound of interest has any similarity with the previously known Hsp modulators. We anticipate that HSPMdb would become a valuable resource for the broader scientific community working in the area of chaperone biology and protein misfolding diseases. HSPMdb is freely accessible at http://bioinfo.imtech.res.in/bvs/hspmdb/index.php

## Introduction

Cellular proteins are exposed to various kinds of stresses such as changes in temperature, pH and metal ion concentrations, which induces protein misfolding and aggregation (1). The intracellular accumulation of protein aggregates adversely affects cell viability and is the underlying basis of various human diseases. Thus, each cell has an evolved set of proteins known as heat shock proteins, which are components of the cellular quality control system to prevent protein aggregation. The heat shock proteins interact with exposed hydrophobic patches of aggregation-prone proteins and thereby protect cells from the deleterious effects of protein aggregates (2,3). These ubiquitously present proteins in different organisms are highly conserved across different species from bacteria to humans. In addition to their role in preventing protein aggregation, Hsps are also involved in protein synthesis, protein trafficking, assembly of multi-protein complexes and protein degradation (4,5).

Based upon the approximate molecular weight, Hsps are categorized into different families such as Hsp100, Hsp90, Hsp70, Hsp60 or Hsp40 family. Hsp100 family of proteins is AAA+ (ATPase associated with diverse cellular activities) superfamily of ATPase that either in coordination with Hsp70 facilitates disaggregation or with a protease rings promotes protein degradation (6). Hsp90 functions to promote refolding of various growth hormone receptors, kinases, transcription factors and many viral proteins (7). Hsp70 functions in coordination with Hsp40s to bind to partially unfolded substrates and promote their folding (2). In addition to stimulating the ATPase activity of Hsp70, Hsp40 also facilitates substrate transfer to the substrate-binding domain of Hsp70s. Hsp70 also binds to the number of other cellular factors that play a crucial role in regulating substrate fate, e.g. interaction with ubiquitin ligase CHIP at C-terminus of Hsp70 promotes substrate degradation. Hsp60 proteins are known to perform variety to functions such as maintenance of mitochondrial protein homeostasis, cellular signalling, and its inactivation is associated with multiple disorders such as in neurodegenerative diseases (8,9). Many of the Hsp families possess highly homologous multiple members which perform both redundant as well as non-redundant functions (10).

Many previous studies have been focused on understanding the mechanism of Hsps action in various biological pathways. To comprehend such enormous data from different studies, few databases have been designed that provide comprehensive understanding of the functions and roles of these chaperones. HSPIR provides information on sequence, structure, localization and biological roles of Hsps (11). A comprehensive information of chaperone interaction could be accessed through Protein Homeostasis Database (12). Similarly, sHSPDb (13) and CrAgDb (14) decipher information about small heat shock proteins and archaeal chaperones respectively.

As various human diseases are related to protein misfolding disorders such as neurodegenerative disorders (15,16), and various forms of cancer (17), Hsps have been extensively studied as potential therapeutic targets against these diseases (18,19), and over the last two decades, considerable efforts have been made towards developing modulators of Hsps activities. Many of these modulators are currently being evaluated for their efficacy in different phases of clinical trials (20–22). However, the information about these modulators from different studies is largely scattered, and no common platform of Hsp modulators with their activities and physicochemical properties has been established until now. Such platform would enable a better understanding of various scaffolds used for targeting different Hsps and thus facilitate rational drug discovery approaches.

In this study, we have made a systematic attempt to collect and compile comprehensive information of experimentally validated modulators (activators and inhibitors) of five major Hsps (Hsp100, Hsp90, Hsp70, Hsp60 and Hsp40) from published literature. The user interface developed in the database also enables users to find the similarity between their compounds of interest with any of the modulators deposited in the database. We anticipate that HSPMdb will be very useful for the scientific community working in the areas of chaperone biology and protein misfolding diseases.

## Materials and methods

### Data collection

All articles available in PubMed were searched to collect and compile comprehensive information on Hsp modulators. To obtain research articles having information on Hsp modulators, systematic searches were performed using various keywords such as ‘heat shock protein modulators’, ‘heat shock protein inhibitors’ and ‘heat shock protein activators’. In addition, the name of individual chaperones such as Hsp100 modulators, Hsp70 modulators and ClpB modulators, was used as a query to search Pubmed articles. These searches ended in a total of 7005 research articles. Articles describing prediction methods, review articles and book chapters were excluded, and the rest of the complete research articles were manually screened by cautious reading for the relevant information of Hsp modulators. Only research papers providing information about experimentally validated Hsp modulators and their analogues were selected for further data curation. Thus, finally 176 research articles were shortlisted and data on Hsp modulators were manually curated. For modulators that have been examined in more than one study or tested against different Hsp types or have been tested by different types of functional assays, multiple entries of such modulators have been made.

### Database architecture and web interface

**Figure 1:**
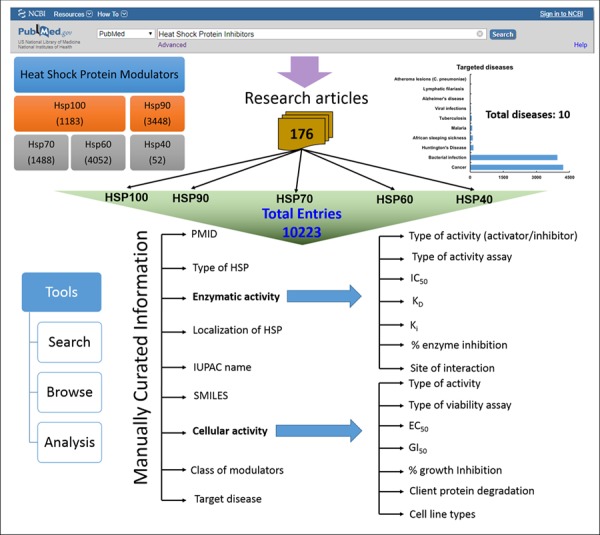
Architecture of HSPMdb.

HSPMdb is built using Linux–Apache–MySQL–PHP (LAMP), a package built on the Linux operating system. LAMP integrates Apache for the web server, and MySQL is a relational database management system. The PHP is scripting language to bring the data fetched by MySQL on to the web pages for display. Additionally, the JAVA script was used to provide dynamic functionalities on web pages. Python scripts are implemented at the back-end to process data fetched from user queries. The overall architecture of HSPMdb is shown in Figure 1.

### Data content

HSPMdb provides comprehensive information on biological and chemical properties of small synthetic Hsp modulators. Biological information of these modulators has been compiled under two major categories: (i) enzymatic activity and (ii) cellular activity. Under the enzymatic activity field, detailed information of the type of enzymatic assay used, site of interaction of modulator, the effect of the modulator and *in vitro* enzymatic modulation activities (IC_50_, EC_50_, DC_50_, EC_50_, *K_i_*, *K*_d_ and percentage inhibition) have been provided. In the cellular activity field, complete information of the type of cell viability assays used, tested cell line and cellular activities (percentage cell growth inhibition, EC_50_ and GI_50_) has been compiled. Comprehensive information of targeted Hsps like their name, origin and localization has also been compiled. Additionally for each compound, the database provides information on 2D/3D structure, and its physical, elemental and topological properties. International Union of Pure and Applied Chemistry (IUPAC) names and Simplified Molecular Input Line Entry System (SMILES) of each modulator were extracted from literatures and further generated using OPSIN (23). The physicochemical properties of all molecules were obtained using the PaDEL software (24) which calculates 2D/3D chemical descriptors from the SMILES of the compounds. The chemical structures of molecules are displayed in the database web pages using online ‘SMILES to image’ tool of RxnFinder (25).

### Implementation of tools

A user-friendly web interface has been developed with various tools for convenience of data searching, browsing and analysis. The description of these tools is given below.

### Search

Two searching options, ‘Simple Search’ and ‘Advanced Search’, have been designed for data searching. Simple search allows users to search for modulators in HSPMdb using their desired keywords related to different fields such as the name of disease or compound or name of Hsp or PMID. A default six fields have been selected for display of the result of the query. Also, users can select various additional fields of their choice for display of results of their query. Advance Search allows users to search HSPMdb with complex queries e.g. more than one type of query at a time by selecting different conditions (e.g. AND & OR) between queries.

### Browsing

**Figure 2:**
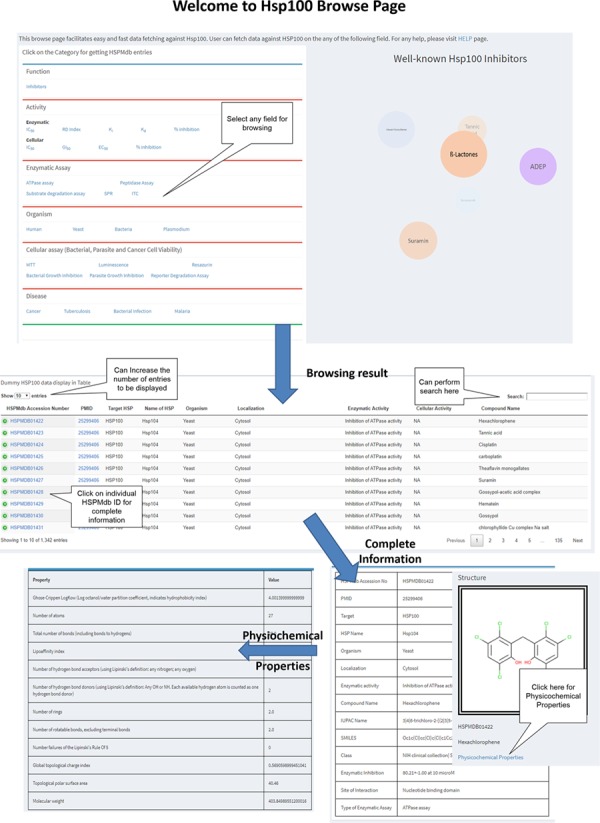
Schematic representation of browsing page of HSPMdb.

To fetch information from HSPMdb, robust browsing pages have been developed (Figure 2). Users can browse on different fields such as enzymatic activity, cellular activity, Disease, Enzymatic and Cellular assays and Organism. In the case of the Enzymatic and Cellular activity field, users can fetch more information on Hsp modulators about its other properties such as IC_50_, EC_50_, DC_50_, EC_50_, *K_i_*, *K*_d_ and percentage inhibition. HSPMdb provides two options for the users. (i) All HSPs: from this browsing page, the user could get information about modulators against all different classes of Hsps for a desired property such as disease-specific or organism-specific. (ii) Individual Hsp: this browsing page will allow the user to fetch information about modulators on a particular Hsp such as Hsp70 or Hsp90.

### Draw compound

The Draw compound is one of the very important tools which allow users to identify Hsp modulators having similarity to their query molecule based on the similarity index. The tool makes use of the JSME editor developed by Bruno Bienfait and Peter Ertl (26). Users need either structure or SMILE of the query molecule to identify modulator(s) having a similar structure(s) in the database. At the back end, the tool compares the user-given SMILES with SMILES of all molecules in the HSPMdb database based on a method which performs fragmentation of SMILES strings into overlapping substrings of a defined size (four in case of this tool) called as LINGOs (27). The similarity is calculated as the Tanimoto coefficient using the number of matching and non-matching LINGOs. The value of the Tanimoto coefficient lies between 0 and 1 with value closer to 1 indicating higher similarity and the value closer to 0 as lower similarity. There are two ways to search for molecules having a structure similar to the query molecule: (i) users can draw the structure of query compound using tools available in the database and get the SMILE, or (ii) users can directly paste the SMILE of the query molecules. By clicking on the ‘Compare with HSPMdb’ button, users will get a list of Hsp modulators showing similarity with the query molecule along with the similarity index. Users can sort the entries by clicking (single and double) on similarity index. Thus, this tool will be very useful to scientific community for repurposing of existing drugs.

## Results and discussion

**Figure 3:**
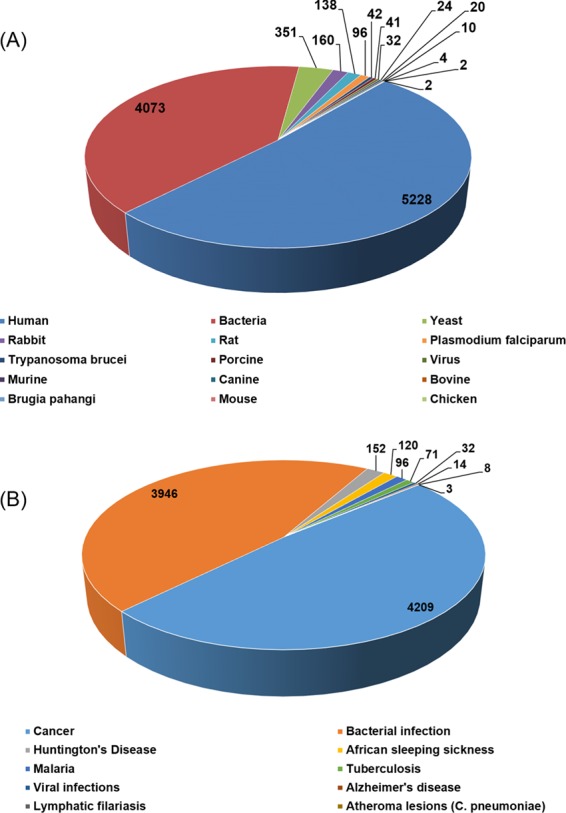
Total number of entries in HSPMdb based upon origin of Hsps (**A**) and targeted diseases (**B**).

The current version of HSPMdb catalogues 10 223 entries of Hsp modulators among which 10 159 entries are of Hsp inhibitors while 59 entries are of Hsp activators. The above information was manually extracted from 176 research articles. HSPMdb provides information of modulators against five different Hsps with maximum entries of Hsp60 (4052 entries) followed by Hsp90 (3448 entries), Hsp70 (1488 entries), Hsp100 (1183 entries) and Hsp40 (52 entries) modulators. These Hsps belong to different organisms (e.g. human, yeast, bacteria, *Plasmodium falciparum*) and currently information about Hsps from a total of 15 organisms has been compiled (Figure 3A). Maximum entries are compiled on modulators against human Hsps (5228 entries) which is as expected from the multiple roles played by these proteins in diverse cellular processes. The presence of a large number of modulators against human Hsps further suggests that targeting these Hsps is one of the major ongoing therapeutic intervention strategies. The second and third most common modulators were found against bacterial Hsps (4073 entries) and yeast Hsps (351 entries) suggesting studies targeting bacterial diseases are more than yeast diseases.

**Figure 4:**
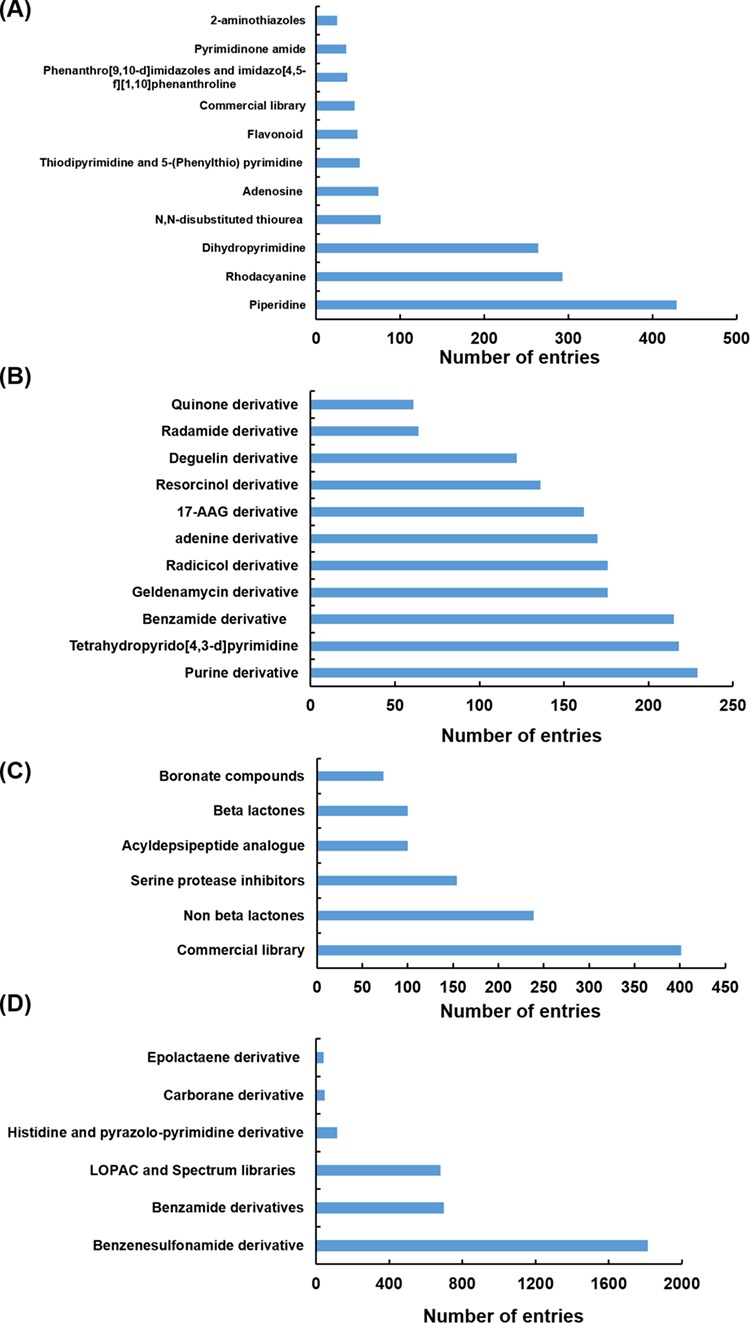
Different scaffolds/classes of modulators targeting Hsp70 (**A**), Hsp90 (**B**), Hsp100 (**C**) and Hsp60 (**D**).

Hsps have been extensively explored as therapeutic targets against various human diseases (18,19,28) primarily associated due to protein misfolding or aggregation. In addition, various pathogens also require their own chaperoning machinery for survival under stressful conditions encountered in the host (29,30). In the current version of HSPMdb, information about Hsps’ modulators against 10 different diseases has been compiled (Figure 3B). We found that most of these modulators are against cancer (4209 entries), followed by bacterial infections (3946 entries). The total number of entries for Hsps’ modulators against cancer, the most widely targeted disease, is 113, 3053, 994 and 49 for Hsp100, Hsp90, Hsp70 and Hsp60, respectively, suggesting Hsp90 is the major target in cancer therapeutics.

The studies mined for the design of current database reports activities of Hsps modulators either in the form of enzymatic activity and/or as cellular activity. The reported enzymatic assays are primarily *in vitro* with purified Hsps and provide information such as IC_50_, *K_i_* and *K*_d_. The cellular-based activity assays are predominantly to examine the effect of modulator on activity of Hsps in a cell-based assay such as measurement of cell-based luminescence or cell growth using MTT (3-(4,5-dimethylthiazol-2-yl)-2,5-diphenyltetrazolium bromide)/Alamar assay. Therefore, experimental data on both activities of Hsp modulators have been collected and reported in the current study. Almost equal entries of modulators for enzymatic (5244) and cellular-based activity assay (4985) have been observed. For enzymatic based activity, we have collected and reported all information about the modulators such as IC_50_, EC_50_, DC_50_, *K_i_*, *K*_d_ and percentage inhibition obtained from various functional assays. In total, information has been compiled from 26 different types of enzymatic assays. Our study shows that the substrate refolding assay is the most widely used assay followed by ATPase assay to examine the effect of molecules on Hsps enzymatic activity. Similarly, in the case of cellular activity, different cellular viability assays like MTT, Alamar blue and resazurin-based assays have been reported in the literature and, thus, we have collected data on such 15 different types of reported cellular assays.

The database reports information from 140 different cell lines used for cell viability assay. The total number of entries of modulators found using cellular viability assay was observed to be 4985. For bacterial growth inhibition assay, 21 different bacterial species have been used resulting in 1594 entries of modulators against various Hsps. For some of the modulators (geldanamycin, MKT-077, MAL3-101, 17-AAG, JG-98), multiple entries have been made as those were examined in multiple studies or tested against different Hsp types or validated by multiple functional/cellular assays.

Hsps are multi-domain proteins, and interaction with other co-chaperones influences their activity. The modulation of Hsps’ activity by various small molecules could be due to their interaction with different regions of the chaperone such as with substrate binding or nucleotide-binding pocket. In addition, many modulators obtained from previous studies have been reported to modulate the activity of Hsps by binding at the interface of the co-chaperone-binding site. To enrich users with such information, we have collected and compiled information of binding site of these modulators on their respective Hsps. We found that most of the modulators bind to the N-terminal domain (5222 entries) while a few (77 entries) were found to interact with the C-terminal domain of Hsps. The dominance of modulators binding to the N-terminal of Hsps suggests that the function of this domain is more sensitive to alteration by the small molecule binders.

Hsp modulators compiled in HSPMdb belong to diverse classes or scaffolds. We observed that in the case of Hsp70 and Hsp90, most of the previous studies had explored the effect of different analogues of already existing modulators (such as of geldanamycin, resorcinol, radicicol, VER155008, YM-08, JG-98 and Apoptozole). For the Hsp100 and Hsp60 family of proteins, studies have primarily reported screening of various available commercial libraries of diverse compounds to identify molecules with modulatory activities. The present database thus provides comprehensive information of different classes/scaffolds of Hsp modulators from a large set of available studies in PubMed (Figure 4). The comprehensive information provided in the present study will facilitate the development of novel inhibitors or activators against various Hsps.

### Summary and future perspectives

HSPMdb will be very useful for a broader scientific community working in the area of chaperone biology and protein misfolding diseases in many ways: (i) the researcher can gather information of all Hsp modulators on a single platform which was not available until now, (ii) users could search HSPMdb for their newly designed molecules to examine whether similar scaffold or identical Hsp modulators have already been reported against any Hsps from 15 different organisms and (iii) HSPMdb provides a novel dataset of a large number of compounds targeting different Hsps which would be useful for developing novel algorithms for the prediction of Hsp modulators.

This is the first version of HSPMdb where information of Hsp modulators has been compiled from available research articles. The only limitation is that information of Hsp modulators from patents have not been incorporated in this version of HSPMdb. Currently, we have compiled information of five major Hsps (Hsp100, Hsp90, Hsp70, Hsp60 and Hsp40), and similar information from small Hsps needs to be provided. The information of small Hsp modulators as well as data from patents will be incorporated in the subsequent updated version of HSPMdb.

## Availability

HSPMdb is available at http://bioinfo.imtech.res.in/bvs/hspmdb/index.php

## References

[1] Hartl F.U. (1996) Molecular chaperones in cellular protein folding. Nature, 381, 571–579. 8637592 10.1038/381571a0

[2] Mayer M.P. and Bukau B. (2005) Hsp70 chaperones: cellular functions and molecular mechanism. Cell Mol Life Sci, 62, 670–684. 15770419 10.1007/s00018-004-4464-6 PMC2773841

[3] Saibil H. (2013) Chaperone machines for protein folding, unfolding and disaggregation. Nat Rev Mol Cell Biol, 14, 630–642. 24026055 10.1038/nrm3658 PMC4340576

[4] Fernández-Fernández M.R. and Valpuesta J.M. (2018) Hsp70 chaperone: a master player in protein homeostasis. F1000Res, 7, F1000 Faculty Rev-1497. 10.12688/f1000research.15528.1 PMC6148205 30338057

[5] Craig E.A., Gambill B.D. and Nelson R.J. (1993) Heat shock proteins: molecular chaperones of protein biogenesis. Microbiol Rev, 57, 402–414. 8336673 10.1128/mr.57.2.402-414.1993 PMC372916

[6] Mogk A., Kummer E. and Bukau B. (2015) Cooperation of Hsp70 and Hsp100 chaperone machines in protein disaggregation. Front Mol Biosci, 2. 10.3389/fmolb.2015.00022 PMC4436881 26042222

[7] Schopf F.H., Biebl M.M. and Buchner J. (2017) The HSP90 chaperone machinery. Nat Rev Mol Cell Bio, 18, 345. 28429788 10.1038/nrm.2017.20

[8] Cheng M.Y., Hartl F.U., Martin J. et al. (1989) Mitochondrial heat-shock protein hsp60 is essential for assembly of proteins imported into yeast mitochondria. Nature, 337, 620–625. 2645524 10.1038/337620a0

[9] Bukau B. and Horwich A.L. (1998) The Hsp70 and Hsp60 chaperone machines. Cell, 92, 351–366. 9476895 10.1016/s0092-8674(00)80928-9

[10] Sharma D. and Masison D.C. (2008) Functionally redundant isoforms of a yeast Hsp70 chaperone subfamily have different antiprion effects. Genetics, 179, 1301–1311. 18562668 10.1534/genetics.108.089458 PMC2475734

[11] R, R.K., N S, N., S P, A., Sinha, D., Veedin Rajan, V.B., Esthaki, V.K. and D'Silva, P (2012) HSPIR: a manually annotated heat shock protein information resource. Bioinformatics, 28, 2853–2855. 22923302 10.1093/bioinformatics/bts520 PMC3476333

[12] Ramakrishnan R., Houben B., Kreft Ł. et al. (2019) Protein homeostasis database: protein quality control in E. coli. Bioinformatics. 10.1093/bioinformatics/btz628 PMC9883681 31392322

[13] Jaspard E. and Hunault G. (2016) sHSPdb: a database for the analysis of small heat shock proteins. BMC Plant Biol, 16, 135–135. 27297221 10.1186/s12870-016-0820-6 PMC4906601

[14] Rani S., Srivastava A., Kumar M. and Goel M. (2016) CrAgDb—a database of annotated chaperone repertoire in archaeal genomes. FEMS Microbiol Lett, 363. 10.1093/femsle/fnw030 26862144

[15] Leak R.K. (2014) Heat shock proteins in neurodegenerative disorders and aging. J Cell Commun Signal, 8, 293–310. 25208934 10.1007/s12079-014-0243-9 PMC4390795

[16] Sharma D. and Masison D.C. (2009) Hsp70 structure, function, regulation and influence on yeast prions. Protein Pept Lett, 16, 571–581. 19519514 10.2174/092986609788490230 PMC2746719

[17] Chatterjee S. and Burns T.F. (2017) Targeting heat shock proteins in cancer: a promising therapeutic approach. Int J Mol Sci, 18, 1978. 10.3390/ijms18091978 PMC5618627 28914774

[18] Kampinga H.H. and Bergink S. (2016) Heat shock proteins as potential targets for protective strategies in neurodegeneration. Lancet Neurol, 15, 748–759. 27106072 10.1016/S1474-4422(16)00099-5

[19] Shirota T., Ojima H., Hiraoka N. et al. (2015) Heat shock protein 90 is a potential therapeutic target in cholangiocarcinoma. Mol Cancer Ther, 14, 1985. 26141945 10.1158/1535-7163.MCT-15-0069

[20] Hendriks L.E.L. and Dingemans A.-M.C. (2017) Heat shock protein antagonists in early stage clinical trials for NSCLC. Expert Opin Inv Drug, 26, 541–550. 10.1080/13543784.2017.1302428 28274158

[21] Jhaveri K., Taldone T., Modi S. and Chiosis G. (2012) Advances in the clinical development of heat shock protein 90 (Hsp90) inhibitors in cancers. Biochim Biophys Acta, 1823, 742–755. 22062686 10.1016/j.bbamcr.2011.10.008 PMC3288123

[22] Kim Y.S., Alarcon S.V., Lee S. et al. (2009) Update on Hsp90 inhibitors in clinical trial. Curr Top Med Chem, 9, 1479–1492. 19860730 10.2174/156802609789895728 PMC7241864

[23] Lowe D.M., Corbett P.T., Murray-Rust P. and Glen R.C. (2011) Chemical name to structure: OPSIN, an open source solution. J Chem Inf Model, 51, 739–753. 21384929 10.1021/ci100384d

[24] Yap C.W. (2011) PaDEL-descriptor: an open source software to calculate molecular descriptors and fingerprints. J Comput Chem, 32, 1466–1474. 21425294 10.1002/jcc.21707

[25] Hu Q.-N., Deng Z., Hu H. et al. (2011) RxnFinder: biochemical reaction search engines using molecular structures, molecular fragments and reaction similarity. Bioinformatics, 27, 2465–2467. 21752802 10.1093/bioinformatics/btr413

[26] Bienfait B. and Ertl P. (2013) JSME: a free molecule editor in JavaScript. J Cheminform, 5, 24–24. 23694746 10.1186/1758-2946-5-24 PMC3662632

[27] Vidal D., Thormann M. and Pons M. (2005) LINGO, an efficient holographic text based method to calculate biophysical properties and intermolecular similarities. J Chem Inf Model, 45, 386–393. 15807504 10.1021/ci0496797

[28] Nahleh Z., Tfayli A., Najm A. et al. (2012) Heat shock proteins in cancer: targeting the ‘chaperones’. Future Med Chem, 4, 927–935. 22571616 10.4155/fmc.12.50

[29] Anup S.R., Dhaval P., Ramya I. et al. (2013) Targeting heat shock protein 90 for malaria. Mini-Rev Med Chem, 13, 1903–1920. 24070209 10.2174/13895575113136660094

[30] Pesce E.R., Cockburn I.L., Goble J.L. et al. (2010) Malaria heat shock proteins: drug targets that chaperone other drug targets. Infect Disord Drug Targets, 10, 147–157. 20334623 10.2174/187152610791163417

